# Particulate air pollution and metabolic risk factors: Which are more prone to cardiac mortality

**DOI:** 10.3389/fpubh.2022.995987

**Published:** 2022-10-20

**Authors:** Erum Rehman, Shazia Rehman

**Affiliations:** ^1^Department of Mathematics, Nazarbayev University, Nur-Sultan, Kazakhstan; ^2^School of Economics, Shandong University of Science and Economics, Jinan, China; ^3^Group of Energy, Economy and Systems Dynamics, University of Valladolid, Valladolid, Spain; ^4^Department of Biomedical Sciences, Pak-Austria Fachhochschule: Institute of Applied Sciences and Technology, Haripur, Pakistan

**Keywords:** CVD, mortality, grey relational analysis, SAARC, risk factors, household air pollution

## Abstract

This study explored multiplex, country-level connections between a wide range of cardiac risk factors and associated mortality within the South Asian Association for Regional Cooperation (SAARC) countries. The grey relational analysis (GRA) methodology is used to evaluate data from 2001 to 2018 to compute scores and rank countries based on cardiac mortality. Subsequently, we used the conservative (Min-Max) technique to determine which South Asian country contributes the most to cardiac mortality. The Hurwicz criterion is further applied for optimization by highlighting the risk factors with the highest impact on cardiac mortality. Empirical findings revealed that India and Nepal are the leading drivers of cardiovascular disease (CVD) mortality among all SAARC nations based on the results of the GRA methodology. Moreover, the outcomes based on the Hurwicz criterion and the conservative criterion indicated that CVD mortality is considerably impacted by household air pollution from the combustion of solid fuel, with India as a potential contributor in the SAARC region. The outcomes of this research may enable international organizations and public health policymakers to make better decisions and investments within the SAARC region to minimize the burden of CVD while also strengthening environmentally sustainable healthcare practices.

## Introduction

Cardiovascular diseases (CVDs) are the leading cause of death and disability worldwide (1). Over the past few decades, cardiac deaths have dropped in various high-income countries (HICs), whereas they have surged in low- and middle-income countries (LMICs), with these economies bearing the majority of the burden, approximately 23% of the world's population (2–4). As a result of rapid modernization, improved survival from acute illnesses, aging, and chronic noncommunicable diseases (NCDs), notably CVDs, have become a grave risk in LMICs, specifically in the densely packed South Asian region (5, 6). In South Asia, NCDs currently contribute to 52% of deaths, and by 2030, they are predicted to be responsible for about 72% of all deaths (7). Ample evidence has demonstrated that South Asians have a higher risk of cardiac disorders, spurring calls to strengthen the management of CVD within this region and among migrant populations (8, 9). In order to establish global and context-specific prevention approaches, we need to document the consistency or discrepancies in the correlations between cardiac risk variables and accompanying deaths, both globally and country-wise categorization by monetary perspectives. To help mitigate the cardiac risk factors at the population level, viable endeavors are recommended.

Multiple objectives are a frequent challenge with systemic issues, which ultimately brings ambiguity. Thus, finding methodologies that incorporate the most criteria in the decision-making procedure and directly affect decisions is important in this situation to decrease inaccuracies (10, 11). The majority of the time, this strategy is challenging to execute due to frequently fluctuating decision-making criteria, increasing the level of uncertainty in the ultimate solution. Health-related procedures are far more complicated since they involve not only technical or financial issues but also a human component that can lead to conflicts of interest and impede the decision-making process. To enhance the health systems overall, several kinds of investigations were performed by applying the multicriteria decision analysis (MCDA) (12, 13). Several distinct MCDA methods conducted in the past in the healthcare domain are summarized in Table 1. These procedures are appreciated for many reasons. From a methodological perspective, researchers hypothesized that the process allowed distinctness, uniformity, and reliability toward more appropriate strategic planning. The MCDA implementation procedures are considered effective and productive in prioritizing contexts more generally (22, 23). These investigations are regulated by employing multiple methodologies to investigate and identify ideal solutions for improving patient care and the healthcare system as a whole. Nevertheless, little is known about the best possible ways to improve the caliber of research and its implementation.

**Table 1:** Multicriteria decision analysis (MCDA) techniques in healthcare.

**No**.	**Research in healthcare using MCDA techniques**	**MCDA Technique**	**Year**	**References**
1	Development, test, and comparison of two multiple criteria decision analysis (MCDA) models: a case of healthcare infrastructure location	ER, AHP	2015	(14)
2	Application of multi-criteria-decision approach for the analysis of medical waste management systems in Myanmar	AHP, ANP	2019	(15)
3	Application of multi-criteria decision approach in the assessment of medical waste management systems in Nigeria	Fuzzy AHP	2021	(16)
4	Evaluating the security impact of healthcare web applications through fuzzy-based hybrid approach of multi-criteria decision-making analysis	Fuzzy AHP-TOPSIS	2020	(17)
5	Measuring the performance of healthcare supply chains in India: A comparative analysis of multi-criteria decision-making methods	MABAC, CoCoSo, MARCOS	2020	(18)
6	Fuzzy-based symmetrical multi-criteria decision-making procedure for evaluating the impact of harmful factors on healthcare information security	Fuzzy AHP-TOPSIS	2020	(10)
7	A multi-criteria decision-making approach for schizophrenia treatment techniques	Fuzzy TOPSIS	2020	(19)
8	**C**apacity evaluation of diagnostic tests for COVID-19 using multicriteria decision-making techniques	Fuzzy PROMETHEE-TOPSIS	2020	(20)
9	Patients' satisfaction and public and private sectors' health care service quality in Pakistan: Application of grey decision analysis approaches	Grey incidence analysis	2016	(21)
10	The use of multi-criteria decision-making models in evaluating anesthesia method options in circumcision surgery	Fuzzy AHP, TOPSIS	2017	(22)
11	A lean approach to healthcare management using multicriteria decision-making techniques	Fuzzy AHP	2021	(23)
12	Identifying a personalized anesthetic with Fuzzy PROMETHEE	Fuzzy PROMETHEE	2020	(24)
13	Comparative evaluation of the treatment of COVID-19 with multicriteria decision-making techniques	Fuzzy PROMETHEE-VIKOR	2021	(25)
14	An extended best-worst multi-criteria decision-making method by belief functions and its applications in hospital service evaluation	BWM-BFT	2020	(26)
15	Fuzzy PROMETHEE for ranking pancreatic cancer treatment techniques	Fuzzy PROMETHEE	2019	(27)
16	A hybrid multi-criteria decision-making method for cloud adoption: Evidence from the healthcare sector	ISM-AHP-TOPSIS	2020	(28)
17	Evaluating nuclear medicine imaging devices using the fuzzy PROMETHEE method	Fuzzy PROMETHEE	2017	(29)
18	Service quality benchmarking via a novel approach based on fuzzy ELECTRE III and IPA: an empirical case involving the Italian public healthcare context	Fuzzy ELECTRE III, IPA	2017	(30)
19	A case-based grey relational analysis model for multiple criteria classification of thyroid nodules.	GIA	2020	(13)
20	Developing a strategic sustainable facility plan for a hospital layout using ELECTRE and Apple's procedure	ELECTRE	2021	(31)
21	Evaluating suppliers for healthcare centers using an ordinal priority approach	OPA	2021	(32)

Particularly as it relates, the relationship between cardiac risk factors and related mortality is being investigated globally using a wide range of scientific and analytical approaches. These studies incorporated either a limited set of risk factors or nations for their investigations and none of them quantitatively scrutinized the role of all South Asian nations, resulting in contradictory outcomes. These bodies of research contributed key insights into the literature studies. However, by considering all South Asian Association for Regional Cooperation (SAARC) economies collectively with a variety of risk factors, we might draw a more comprehensive breadth of the connections. Accordingly, in the current investigation, we attempted to fill the gaps in the literature by probing the nexus between low physical activity, high blood pressure (hypertensive), smoking, high body-mass index (BMI), high fasting plasma glucose (diabetes mellitus), high total cholesterol (hyperlipidemia), household air pollution from combustion of solid fuels, ambient air pollution, and cardiac mortality within the SAARC region (Maldives, Bangladesh, India, Afghanistan, Nepal, Sri Lanka, Pakistan, and Bhutan) using the grey relational models (GRA) models: (i) Deng GRA, (ii) absolute GRA, and (iii) the second synthetic GRA (SSGRA) model. The purpose of the absolute GRA model is to provide integral closeness or proximity between two variables, whereas Deng's GRA model just reveals partial closeness or proximity. The second synthetic GRA model, on the other hand, incorporates the characteristics of both Deng's GRA and absolute GRA models to offer a more comprehensive closeness, also known as inclusive proximity. This closeness or proximity is also known as the correlation in the current literature. The core idea behind the GRA models is to evaluate the degree of relationships between the study variables based on how comparable their geometric curves are to one another. To put it simply, the fundamental goal of GRA models is to quantify the proximity or closeness of two data sequences that indicate two curves from a distinct perspective. These insights set one GRA model apart from another.

**Figure 1:**
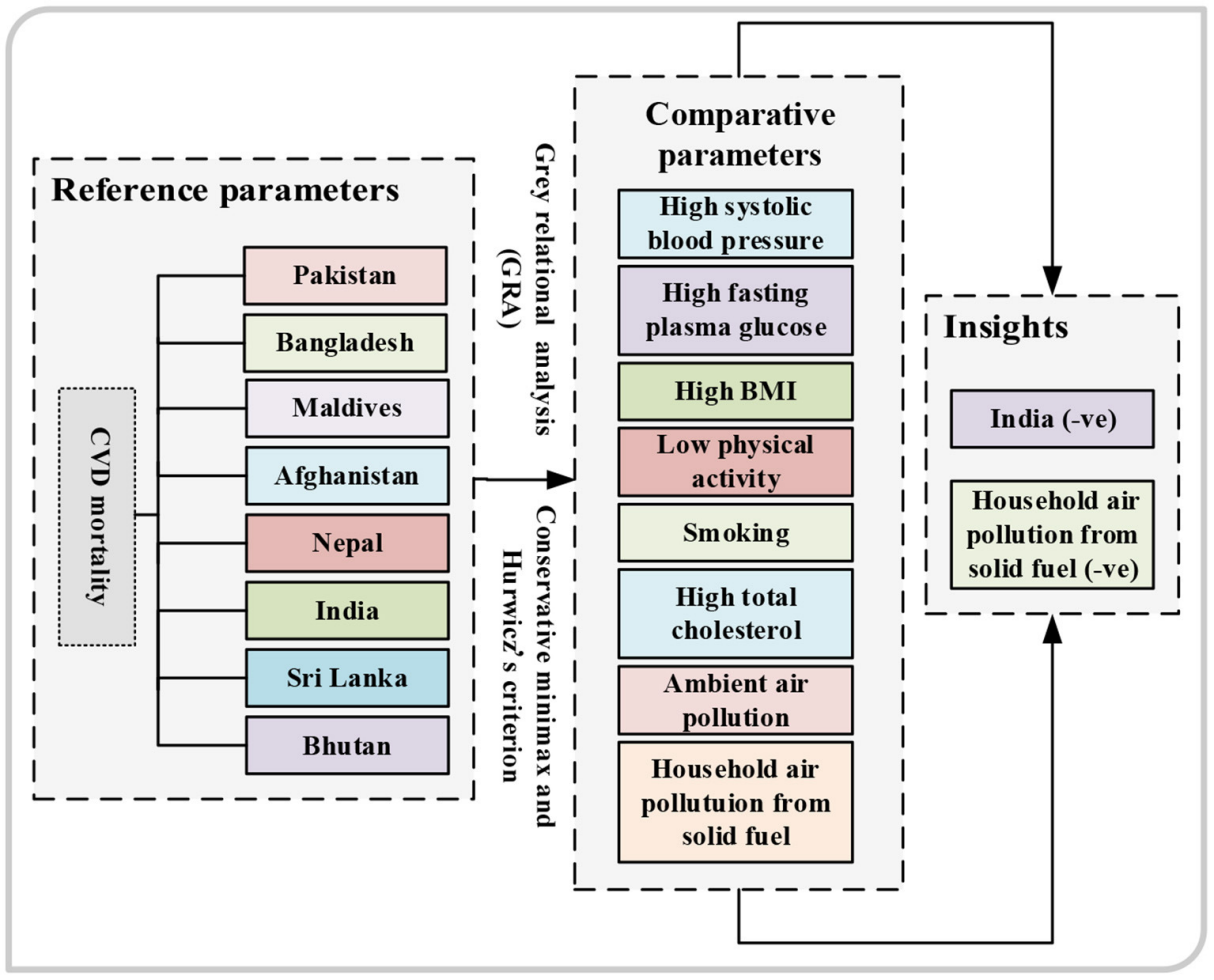
The graphical abstract.

Based on the notion of an association between the reference series and the comparability series of the data, GRA can ascertain the potential factors of the given inputs. These models have higher precision and could produce more reliable solutions only with limited data. Besides, we also utilized two decision-making approaches: (i) the Hurwicz (Min-Max) criterion and (ii) the conservative criterion to carry out a comparative assessment of all risk factors and cardiac mortality within South Asia to indicate the significant contributor (among country and chosen risk factors) of cardiac mortality. When compared to existing strategies for compelling outcomes, the recommended approaches are more effective and help to minimize endogeneity challenges. The proposed model is a vital tool and an additional pragmatic framework for policymakers who seek to mitigate mortality attributable to cardiac events. Figure 1 represents the graphical framework of the investigated work.

## Materials and methods

### Data source

Data on the prevalence of cardiac risk factors (high systolic blood pressure, high fasting plasma glucose, high BMI, low physical activity, smoking, high total cholesterol, ambient air pollution, household air pollution due to combustion of solid fuels, and cardiac mortality within the SAARC nations (Afghanistan, Bangladesh, India, Maldives, Nepal, Sri Lanka, Bhutan, and Pakistan) are retrieved from the Global Burden of Disease (GBD) Study 2018 for the timespan 2001–2018.

### Grey relational analyses

**Figure 2:**
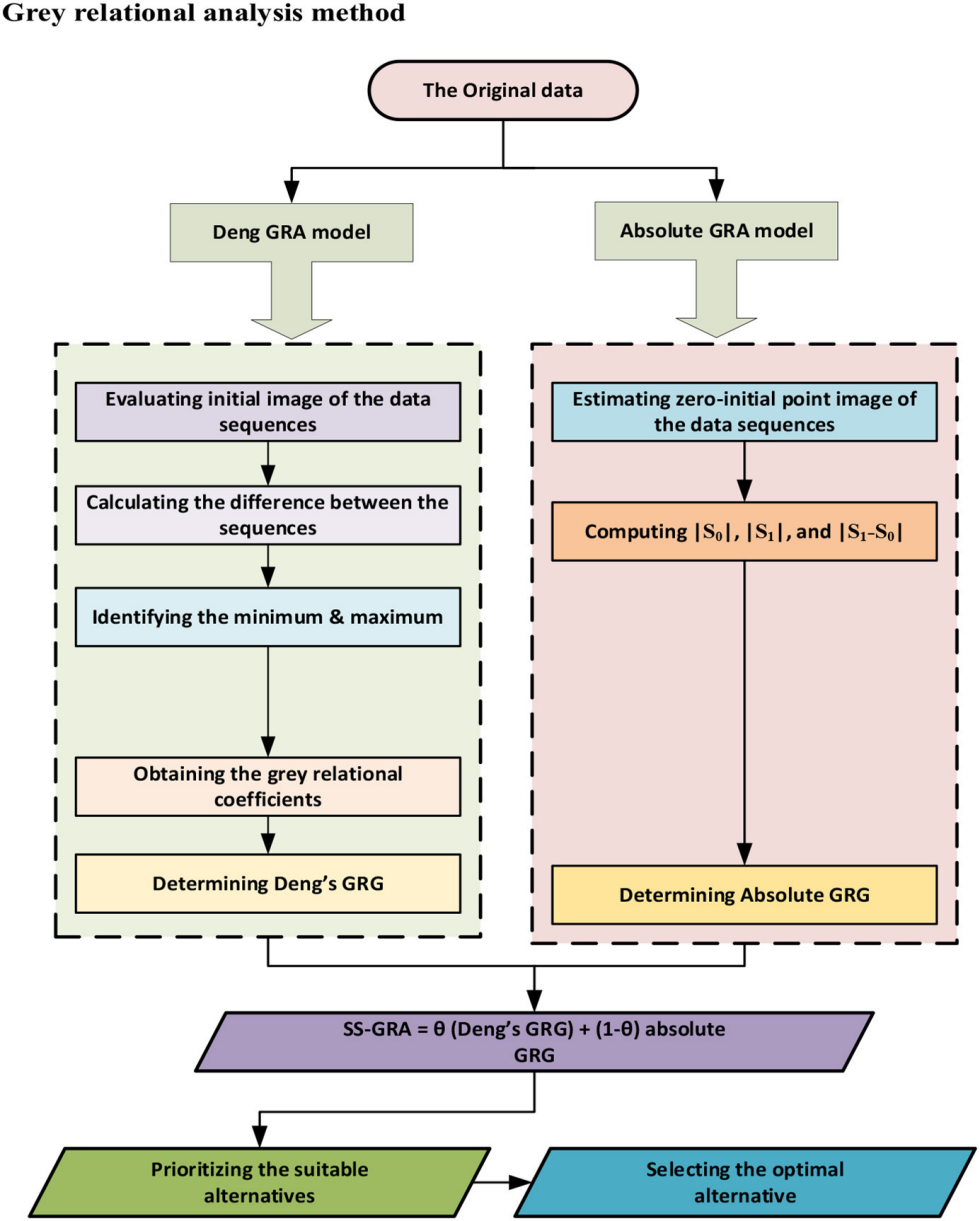
The operationalized framework of grey relational analyses (GRA).

Grey relational analysis methods are one of the fundamental aspects of grey system theory (GST), which was presented in 1982 by Chinese scholar Deng Julong to deal with ambiguous processes with partial information (33). The basic principle behind grey modeling is that the measure of closeness (or correlation) of the multilateral pattern of a given dataset representing the structural properties could be used to foresee the closeness of a linkage among system variables. A detailed description of the GRA method can be found in Liu et al.'s study (34). The grey methodology was successfully applied in a variety of research domains (35–39). The data series was investigated by engaging GRA methodologies. The GRA models were designed using SPSS (2019), whereas Microsoft Excel software (2019) was used to solve the conservative (min-max) criterion and the Hurwicz's criterion. Initially, the degree of correlations is estimated between the chosen study variables independently using Deng's degree of GRA, and then, the degree of influence is estimated using the absolute degree of the GRA model. Later, the second synthetic GRA model is estimated by incorporating the outcomes obtained from Deng GRA and absolute GRA models, which we named weights. Based on those calculated weights, we set a ranking pattern of the countries to conclude which country is more prone to the selected risk factors. For decision-making under uncertainty, the conservative (Min-Max) technique is deployed to choose the most influential factor affecting cardiac mortality within South Asia. Furthermore, Hurwicz's approach is adopted to distinguish the risk variables that have a relatively strong influence on CVD mortality in South Asian countries. Figure 2 depicts the conceptualized GRA paradigm. The accompanying section summarizes the computing algorithms for grey relational models.

#### Deng's GRA model

Let *Y*_*i*_ be the reference sequence denoting a dependent parameter and *Y*_*j*_ be the comparative sequences denoting an independent parameter. Then, at that point grey relational gradient (GRG), the real number degree addressing the output of the GRA model is depicted as γ_*ij*_ *or γ*(*𝕐*_*i*_, *𝕐*_*j*_) and can be accompanied by:


$$\gamma \left(Y_{i}\;,\;{𝕐}_{j}\right)=\;\frac{1}{\hbar }\sum_{l=1}^{\hbar }\gamma \left(Y_{i\left(\;\ell \right)},Y_{j\left(\;\ell \right)}\;\right),$$

Where,


$$\begin{array}{l} \;\gamma \left(Y_{i\left(\ell \right)},Y_{j\left(\ell \right)}\right)\\ =\;\frac{\min_{k}\min_{\ell }\left|Y_{i\left(\ell \right)}-Y_{j\left(\ell \right)}\right|+\zeta \;\max_{k}\max_{\ell }\left|Y_{i\left(\ell \right)}-Y_{j\left(\ell \right)}\right|}{\left|Y_{i\left(\ell \right)}-Y_{j\left(\ell \right)}\right|+\;\zeta \;\max_{k}\max_{\ell }\left|Y_{i\left(\ell \right)}-Y_{j\left(\ell \right)}\right|} \end{array}$$

In this case, ζ* ϵ* (0, 1) represents a distinguishing coefficient and is assumed to be at 0.5.

#### Absolute GRA model

If *𝕐*_*i*_(*dependent*) *and 𝕐*_*j*_(*independent*) are two distinct data series connected within a system, the algorithm for computing the absolute GRA is shown below (34).


$$\begin{array}{l} \epsilon_{ij}=\frac{1+|r_{i}|+|r_{j}|}{1+|r_{i}|+|r_{j}|+|r_{i}-r|} \end{array}$$

$$r_{i}=\;\int_{1}^{\hbar }{𝕐}_{i}^{0}dt,\;r_{j}=\int_{1}^{\hbar }{𝕐}_{j}^{0}dt,\;r_{i}-\;r_{j}=\;\int_{1}^{\hbar }\left({𝕐}_{i}^{0}-\;{𝕐}_{j}^{0}\right)\;dt$$

#### Second synthetic GRA model

The Second synthetic GRA (SSGRA) model can be produced by incorporating the given equation.


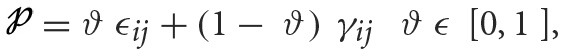


where ′
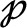
′ indicates the SSGRA model, ${}^{\prime }\epsilon_{ij}^{\prime }$ indicates the absolute GRA model, and ′γ′ indicates Deng's GRA model outcomes across the two grey data series, i.e., Y_i_ and Y_j_. Whenever a decision-maker seeks an inclusive evaluation that combines the pros of both ′ϵ′and ′γ′ without endorsing one over another and so sets ϑ at 0.5, we assumed ϑ* at* 0.5 for our analysis. The detailed literature on grey modeling can be obtained from Liu et al. and Kalyon et al.'s studies (34, 40).

## Results

**Figure 3:**
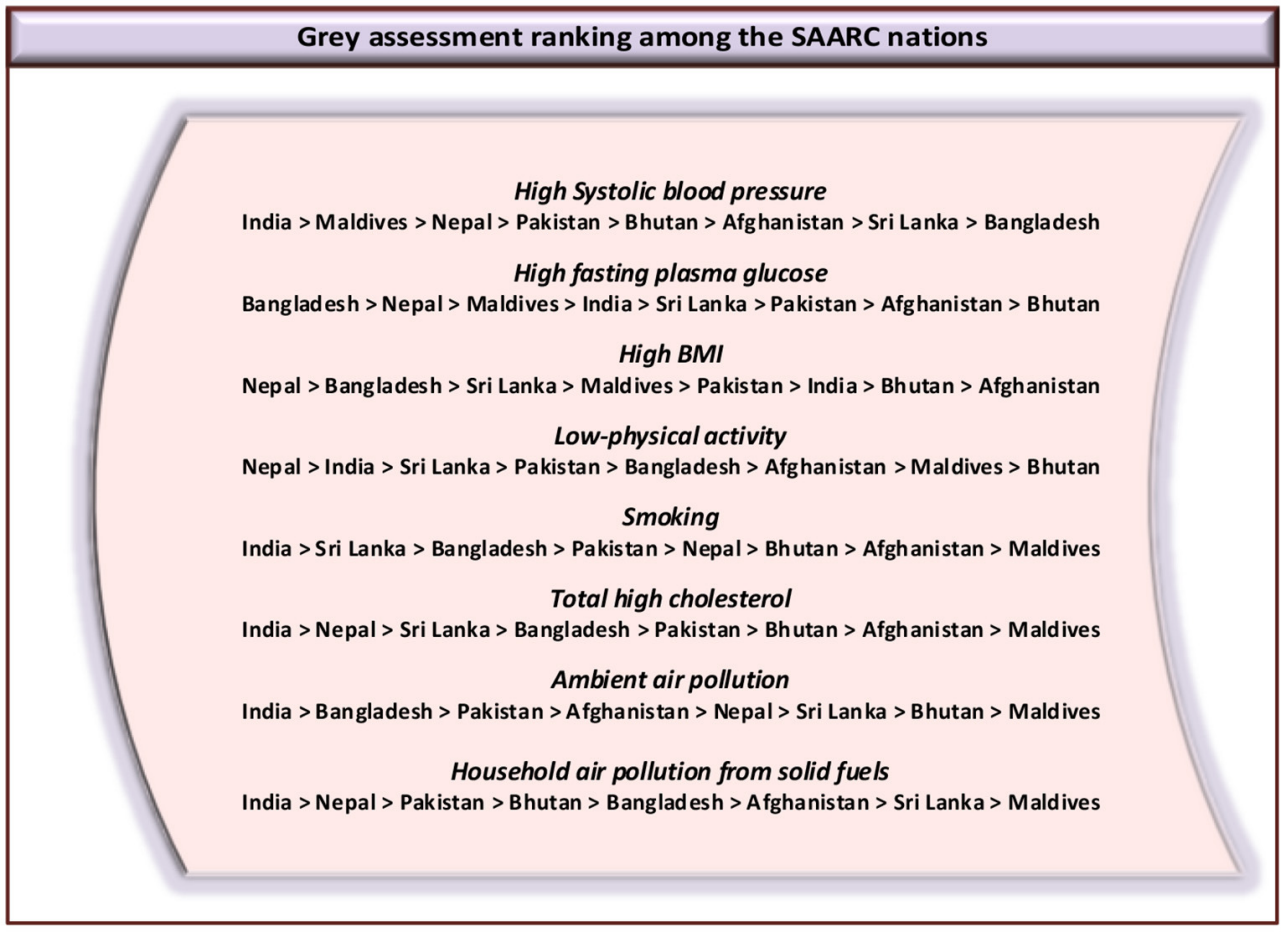
Ranking sequence based on grey assessment.

The present study employed grey techniques to accurately measure the degree of relationship between cardiac risk factors and related mortality within SAARC economies (Afghanistan, Bangladesh, India, Maldives, Nepal, Sri Lanka, Bhutan, and Pakistan) from 2001 to 2018. Table 2 shows the conclusions of the grey relational models, specifically the Deng GRA, the Absolute GRA, and the SSGRA, for cardiac risk factors and associated mortality among SAARC economies. The parameters for the SSGRA and absolute GRA models range from 0–1, while, in the case of Deng's GRA model, it ranges from 0.5–1. Additionally, it is regarded to be strongly associated if it is close to 1 and frailly assosciated if it deviates from 1. The obtained ranking sequence based on GRA is shown in Figure 3 in decreasing sequence for comparison purposes. Table 3 displays the criterion action matrix of GRA.

**Table 2:** Grey assessment between cardiac risk factors and associated mortality.

**Country**	**Absolute GRA**	**Deng GRA**	**SSGRA**
Pakistan	0.9458	0.6370	0.7914
Bangladesh	0.7103	0.7393	0.7248
Maldives	0.8004	0.9212	0.8608
Afghanistan	0.7846	0.7270	0.7558
Nepal	0.8097	0.8855	0.8476
India	0.9785	0.8195	0.8990
Sri Lanka	0.8258	0.6350	0.7304
Bhutan	0.8945	0.6459	0.7702
India < Maldives < Nepal < Pakistan < Bhutan < Afghanistan < Sri Lanka < Bangladesh			
Pakistan	0.9127	0.6889	0.8008
Bangladesh	0.9186	0.8896	0.9041
Maldives	0.9339	0.7877	0.8608
Afghanistan	0.8935	0.6381	0.7658
Nepal	0.9632	0.7944	0.8788
India	0.9301	0.7437	0.8369
Sri Lanka	0.9069	0.7569	0.8319
Bhutan	0.8043	0.6799	0.7421
Bangladesh < Nepal < Maldives < India < Sri Lanka < Pakistan < Afghanistan < Bhutan			
Pakistan	0.9187	0.6791	0.7989
Bangladesh	0.9785	0.7171	0.8478
Maldives	0.8550	0.7458	0.8004
Afghanistan	0.5969	0.7008	0.6488
Nepal	0.9738	0.8268	0.9003
India	0.8552	0.6818	0.7685
Sri Lanka	0.9659	0.7149	0.8404
Bhutan	0.6764	0.7280	0.7022
Nepal < Bangladesh < Sri Lanka < Maldives < Pakistan < India < Bhutan < Afghanistan			
Pakistan	0.9015	0.8007	0.8511
Bangladesh	0.8628	0.8110	0.8369
Maldives	0.6917	0.5983	0.6450
Afghanistan	0.8686	0.6678	0.7682
Nepal	0.9356	0.8766	0.9061
India	0.9618	0.7992	0.8805
Sri Lanka	0.9482	0.8114	0.8798
Bhutan	0.6406	0.5748	0.6077
Nepal < India < Sri Lanka < Pakistan < Bangladesh < Afghanistan < Maldives < Bhutan			
Pakistan	0.9748	0.7668	0.8708
Bangladesh	0.9494	0.7998	0.8746
Maldives	0.7039	0.7001	0.7020
Afghanistan	0.8140	0.6956	0.7548
Nepal	0.8383	0.8547	0.8465
India	0.9276	0.8840	0.9058
Sri Lanka	0.9147	0.8555	0.8851
Bhutan	0.8400	0.8202	0.8301
India < Sri Lanka < Bangladesh < Pakistan < Nepal < Bhutan < Afghanistan < Maldives			
Pakistan	0.9007	0.7985	0.8496
Bangladesh	0.8731	0.8369	0.8550
Maldives	0.6501	0.6101	0.6301
Afghanistan	0.7021	0.6993	0.7007
Nepal	0.9760	0.8214	0.8887
India	0.9366	0.8590	0.8978
Sri Lanka	0.9313	0.8223	0.8768
Bhutan	0.7892	0.7028	0.7460
India < Nepal < Sri Lanka < Bangladesh < Pakistan < Bhutan < Afghanistan < Maldives			
Pakistan	0.8805	0.8685	0.8745
Bangladesh	0.9735	0.8257	0.8996
Maldives	0.7430	0.6350	0.6890
Afghanistan	0.7952	0.7666	0.7809
Nepal	0.7900	0.7440	0.7670
India	0.9537	0.8477	0.9007
Sri Lanka	0.7983	0.6991	0.7487
Bhutan	0.7761	0.7007	0.7384
India < Bangladesh < Pakistan < Afghanistan < Nepal < Sri Lanka < Bhutan < Maldives			
Pakistan	0.8559	0.7679	0.8119
Bangladesh	0.6989	0.7115	0.7025
Maldives	0.4831	0.5171	0.5001
Afghanistan	0.6709	0.5695	0.6202
Nepal	0.9659	0.8001	0.8830
India	0.8983	0.8819	0.8901
Sri Lanka	0.5160	0.6980	0.6070
Bhutan	0.7666	0.7634	0.7650
India < Nepal < Pakistan < Bhutan < Bangladesh < Afghanistan < Sri Lanka < Maldives			

Dependent variable: cardiovascular mortality; independent variables: all selected risk factors.

**Table 3:** Criteria action matrix.

**SSGRA**	**C_1_**	**C_2_**	**C_3_**	**C_4_**	**C_5_**	**C_6_**	**C_7_**	**C_8_**
A_1_	0.7914	0.7248	0.8604	0.7558	0.8476	0.8990	0.7304	0.7702
A_2_	0.8008	0.9041	0.8608	0.7658	0.8788	0.8369	0.8319	0.7421
A_3_	0.7989	0.8478	0.8004	0.6488	0.9003	0.7685	0.8404	0.7022
A_4_	0.8511	0.8369	0.6450	0.7682	0.9061	0.8805	0.8798	0.6077
A_5_	0.8708	0.8746	0.7020	0.7548	0.8465	0.9058	0.8851	0.8301
A_6_	0.8496	0.8550	0.6301	0.7007	0.8887	0.8978	0.8768	0.7460
A_7_	0.8745	0.8996	0.6890	0.7809	0.7670	0.9007	0.7487	0.7384
A_8_	0.8119	0.7025	0.5001	0.6202	0.8830	0.8901	0.6070	0.7650

According to Absolute GRA findings, India showed the highest linkage between high systolic blood pressure and cardiac deaths, whereas a frail association is experienced in Bangladesh. Similarly, a distinct sequence is generated among SAARC nations based on Deng's GRA model. Maldives accumulated the maximum weight accompanied by Nepal and Bangladesh based on the results of Deng's GRA model. The weakest associations were found in Sri Lanka and Pakistan, with grey scores of 0.5761 and 0.6370, respectively. Consequently, the SSGRA model projections revealed that, among South Asian economies, India appeared to be the strongest country for raising CVD mortality, owing to raised blood pressure, trailed by Maldives and Nepal. Hypertension influenced approximately 22% of the overall population and is thought to be the cause of around 9.4 million deaths each year (41). As per the reports of the World Health Organization (WHO), high blood pressure is more concerning in LMICs, and India is not an exception, as it is currently experiencing a 2-fold disease burden because it transitioned from a period of communicable diseases to one with a higher prevalence of NCD (42). The substantial connection between high systolic blood pressure and CVD mortality in India emphasized that there is an immediate need for hypertension prevention and efforts to optimize the hypertension care continuum.

A multitude of detrimental health and cognitive effects are coupled with high fasting plasma glucose and CVDs (43). Nepal is recognized as the SAARC country with the most substantial contribution to cardiac mortality due to high fasting plasma glucose with a grey score of 0.96321; however, Bhutan has the least favorable association under absolute GRA model output. Additionally, the strength of association for Nepal is shown to be significant as compared to Bhutan. Nonetheless, as per Deng's GRA model, Bangladesh gave off an impression of being profoundly powerful in raising CVD mortality and positioned top among SAARC nations, whereas Afghanistan showed up with the most fragile relationship by acquiring a weight of 0.6381 and positioned last. Notably, with the SSGRA model, Bangladesh sustained its position and placed first within all South Asian economies, demonstrating that the country is significantly potent in raising CVD mortality, owing to high fasting plasma glucose levels in its population, trailed by Nepal and Maldives. Afghanistan and Bhutan were found to have the least effect on cardiac mortality in the SAARC region by attaining grey scores of 0.7658 and 0.7421, respectively. The increased level of fasting plasma glucose is related to an increased incidence of DM, which is a substantial risk factor in the progression of cardiovascular disorders. Our findings corroborated some earlier investigations that showed a substantial linkage between high fasting plasma glucose levels and higher CVD mortality (44, 45). This finding emphasized that the significance of raising awareness and screening together with a customized approach is fundamental for ending the scourge of diabetes and pre-diabetes in Bangladesh.

The literature provided abundant evidence that the predominance of high BMI is reliably and firmly related to a greater risk of cardiac incidence and in turn leading to mortality globally (46, 47). As per the results of Deng's GRA model, Bangladesh showed a more stable degree of association between high BMI and cardiac mortality while Afghanistan showed the weakest connection. In the case of Nepal, the findings appeared to be the same for Deng GRA and the SSGRA models, signifying a significant linkage between cardiac-related mortality and high BMI in its population led by Bangladesh and Sri Lanka. The significant linkage between high BMI and cardiac deaths in Nepal was revealed to be the most striking risk factor for assessing and foreseeing cardiac-related mortality. Afghanistan and Bhutan emerged as the SAARC economies with the least grey scores, representing the frail relationship of high BMI with cardiac mortality under absolute and SSGRA model outcomes, individually. The outcomes supported earlier studies that established the relevance of high BMI in the onset and progression of cardiac-related diseases (48, 49). Obesity will disproportionately affect LMICs in the future. Continuous urbanization stimulates risk factors such as sedentary behavior and fat and sugar-loaded eating regimens. Low-income nations like Nepal experience a 2-fold disease burden of communicable dieases and NCDs, which are usually accompanied by obesity (50).

Considering the impact of low physical activity on cardiac mortality within the South Asian region, Pakistan, Maldives, and Bhutan sustained their positions against all grey relational models. However, the degree of relationship was assessed to be more significant for the Pakistani populace than for Maldives and Bhutan. Sri Lanka positioned second under absolute GRA and Deng GRA models, but moved its position to third under the SSGRA model; however, the estimated grey relational weight is found higher in absolute GRA. Similarly, among all SAARC countries, the assessed outcomes for Afghanistan remained the same under Deng GRA and the SSGRA models with a weak intensity of association. Under the SSGRA model, the strength of the relation is meaningfully stronger for Nepal, suggesting that low physical activity has a sizeable influence on mortality in its dense population, accompanied by India and Sri Lanka. The grey relational findings of our study are found reliable with a scope of past investigations in which sedentary behavior is discussed as a significant risk factor. As the cardiac burden has been expanding, primary and secondary care are likely to be fundamental public healthcare policy priorities in the foreseeable future (51, 52).

Based on the findings from the Deng GRA and SSGRA models with relatively stronger intercorrelations, smoking was found to be the most significant prognostic risk factor for cardiac mortality in the Indian population. Nevertheless, Bangladesh and Sri Lanka were ranked second and third, respectively, in the South Asian region. As determined by the absolute GRA and SSGRA models, Maldives and Afghanistan had the lowest scores, suggesting that they have the lowest disease burden among SAARC nations as a result of smoking. Considerable studies showed that smoking raises the risk of almost all CVD subtypes, essentially increasing the risk of cardiac mortality, such as stroke, heart failure, and AMI (53, 54). It is estimated that, currently, 275 million individuals consume tobacco in India. Tobacco is projected to cause approximately one million deaths per annum in India, posing a significant burden of mortality (55). Treatments for smoking cessation that are based on scientific evidence are available. To combat the tobacco pandemic, more dissemination, adoption, and execution are essential. The cardiology department has a professional responsibility to help improve tobacco control initiatives and can play a pivotal role in accomplishing a smoke-free future.

Table 2 provides an analysis utilizing the grey relational models of the association between high total cholesterol and mortality associated with cardiac disorders within South Asia. The findings of this research corroborated with past research studies that established the importance of high total cholesterol with cardiac events and related mortality. Also, given the scores computed by each of the three grey relational models, Sri Lanka, Bhutan, Afghanistan, and the Maldives continued their position and were designated as third, sixth, seventh, and eighth, sequentially. Despite this, Maldives and Afghanistan are considered the least influential countries with the impact of high total cholesterol on CVD mortality among South Asian countries. It is important to note that Nepal obtained the highest weight based on the calculated findings of the SSGRA model portraying the contribution of the significant burden of disease in its populace due to high total cholesterol trailed by India and Sri Lanka. Epidemiological and clinical research exhibited that an abnormal degree of cholesterol intensifies the risk of coronary heart disease (CHD) (56, 57). Evidence also suggested that having low HDL-C and high triglyceride levels were a substantial risk factor for major coronary events (58), and the *post hoc* analysis of various clinical trials revealed that individuals with low HDL-C and high triglycerides levels have the highest likelihood of suffering from CHD (59). To minimize or forestall the risk of CVD in the Nepalese population, the promotion of a healthy lifestyle and the implementation of a comprehensive elevated serum cholesterol screening, treatment, and control plan were required.

Our study additionally explored the influence of outdoor pollution on CVD mortality among SAARC nations. The conclusions of GRA models revealed that Bangladesh was positioned at the top under the absolute GRA model, Pakistan ranked first under Deng's GRA model, whereas India ranked top under the SSGRA model, which indicated a potential effect of ambient air pollution as a substantial predictive risk factor of CVD mortality. Furthermore, it appeared that Sri Lanka, Bhutan, and the Maldives were the countries with the weakest evidence of a link between outdoor pollution and cardiac mortality. Similarly, Bangladesh ranked second under the Deng GRA and SSGRA model outcomes while Pakistan was positioned third under the absolute GRA and SSGRA model outcomes, depicting that, after India, these regions had the most notable repercussions of ambient air pollution on cardiac mortality, with a strong linkage. The current findings supported several other research studies that demonstrated a significant contribution of ambient air pollution to the onset, progression, and mortality of CVD.

An intriguing ranking pattern emerged when the effect of household pollution due to solid fuels on cardiac mortality was examined among SAARC economies. The risk factor of household air pollution signified a greater impact on CVD deaths in densely populated countries of Nepal and India, although they shuffled their position under the absolute and Deng GRA models. The findings exhibited that household air pollution is a significant prognostic risk factor to measure and foresee cardiac event-related fatalities among the population of Nepal and India. Pakistan, Bangladesh, Bhutan, and the Maldives maintained their ranking order against each of the three grey relational outcomes with a significant degree of correlation observed in the case of Pakistan in particular. Of note, Afghanistan ranked sixth and Sri Lanka ranked seventh under the absolute and SSGRA models, depicting the least influence of the disease burden because of indoor pollution due to solid fuels. The findings of the present study showed a high correlation between indoor pollution due to solid fuels and several cardiac ailments and related mortality, which was consistent with the range of epidemiological studies (39, 60, 61).

To proceed with the further analysis, we defined the country variables as Pakistan (C_1_), Bangladesh (C_2_), Maldives (C_3_), Afghanistan (C_4_), Nepal (C_5_), India (C_6_), Sri Lanka (C_7_), and Bhutan (C_8_). However, risk factors are defined as high systolic blood pressure (A_1_), high fasting plasma glucose (A_2_), high BMI (A_3_), low physical activity (A_4_), smoking (A_5_), total high cholesterol (A_6_), ambient air pollution (A_7_), and household air pollution from solid fuel (A_8_).

### The conservative (min-max) criterion

Subsequently, we followed the conservative (Min-Max) technique to ascertain which South Asian economy is more accountable for the rise in cardiac mortality. This metric was adopted in line with the guidance provided by Prasad et al. (62). As cardiac mortality needs to be lowered, the following Min-Max criterion was adopted:


$$\text{min }C_{p}\left\{\max A_{k}\;v\;(A_{k},C_{p})\right\}=\text{min }C_{p}\left\{\begin{array}{l} 0.8990\\ 0.9041\\ 0.9003\\ 0.9061\\ 0.9058\\ 0.8987\\ 0.9007\\ 0.8901 \end{array}\right\}=0.8901$$

According to the estimated figures, among the SAARC countries, India has a substantial part attributable to cardiac events. The results of the decision-making procedure demonstrated that India can promote a novel approach and implemented corrective actions to lower total cardiac mortality within South Asia.

### Hurwicz's criterion result

Hurwicz's method is applied to indicate probable CVD mortality risk factors among SAARC countries. The reference can be seen in the literature on the algorithms of this method (63). Hurwicz's criterion would be determined by maintaining α = 0.8 since we seek to reduce cardiac mortality for the present investigation.


$$\begin{array}{l} \min A_{k}\left\{\alpha \;min\;C_{p}\;v\;\left(A_{k},C_{p}\right)+\left(1-\alpha \right)\;max\;C_{p}\;v\;\left(A_{k},C_{p}\right)\right\} \end{array}$$

Table 4 displays the weighted mean that is determined using Hurwicz's criterion. Given the estimated weighted average, indoor pollution due to combustion (0.5781) was revealed to be a more intense risk factor for accelerated cardiac mortality among SAARC countries when compared with the remainder of the risk determinants. The projected findings of both decision-making techniques demonstrated that, among SAARC nations, Nepal is the leading contributor to cardiac mortality, while household air pollution is the most intensified risk factor in accelerating cardiac events. Thus, renewed endeavors to address the prevention, intervention, diagnosis, and administration of CV risk factors among SAARC countries are required.

**Table 4:** Assessment based on the Hurwicz criterion.

**Max**	**Min**	**Weighted average**	**Hurwicz's decision**
0.8990	0.7248	A_1_ [(0.8 × 0.7248) + (0.2 × 0.8990)] = 0.7596	
0.9041	0.7421	A_2_ [(0.8 × 0.7421) + (0.2 × 0.9041)] = 0.7745	
0.9003	0.6488	A_3_ [(0.8 × 0.6488) + (0.2 × 0.9003)] = 0.6991	
0.9061	0.6077	A_4_ [(0.8 × 0.6077) + (0.2 × 0.9061)] = 0.6674	
0.9058	0.7020	A_5_ [(0.8 × 0.7020) + (0.2 × 0.9058)] = 0.7428	
0.8987	0.6301	A_6_ [(0.8 × 0.6301) + (0.2 × 0.8987)] = 0.6838	
0.9007	0.6890	A_7_ [(0.8 × 0.6890) + (0.2 × 0.9007)] = 0.7313	
0.8901	0.5001	A_8_ [(0.8 × 0.5001) + (0.2 × 0.8901)] = 0.5781	Minimum

## Discussion

The findings of GRA revealed that India and Nepal are the leading drivers of high CVD mortality in the regions of South Asia. Of note, Afghanistan, Bhutan, and the Maldives appeared as the least influential countries in terms of disease burden in the present investigation. Also, the outcomes of the decision-analysis integrating two distinct approaches demonstrated a robust connection between India, household air pollution from solid fuels, and cardiac mortality within the SAARC economies. More precisely, the findings of the present investigations indicated that household air pollution from solid fuels significantly affected CVD mortality, with India as a leading contributor of CVD-related deaths in the SAARC regions more potentially than other variables.

The primary contribution of the present study is that it takes into account a range of determinants and mortality associated with CVD in all SAARC nations. In India, the prevention of risk factors to forestall the CVD burden is poor. Although patients with such ailments need medical care, they are commonly misdiagnosed, overlooked, or left unmanaged. To curb the rise of the CVD burden in India, new cost-effective techniques to alter cardiac risk factors will need to be implemented, distributed, scaled, and sustained (64, 65). Developed economies encountered considerable reductions in age-adjusted cardiac mortality because of population-wide primary preventive efforts combined with advances in secondary and emergency treatment. To accomplish comparable additions, India must adopt the populace-level approach intercessions while fortifying and coordinating its regional, local, and national health care systems. To enable all Indians to achieve their right to health, providing universal health insurance, particularly financial risk protection, remains an idealistic objective. Innovation in research across the therapeutic continuum will be fundamental for effective control and prevention of CVD in India, with bits of knowledge that could impact worldwide endeavors.

Surprisingly, of all selected CV risk factors, household air pollution held considerable domination in cardiac deaths within South Asian countries. Chronic exposure to air pollutants is a known risk factor for a range of CV-associated morbidity and mortality (66, 67). Almost 33% of the total population and an incredibly greater part of households in low-income settings depend on biomass fuels like wood, coal, manure, and agricultural wastes as their principal source of homegrown energy. Because of their fragmented ignition, a large number of pollutants related to undeniable degrees of indoor air pollution are produced, which incorporate suspended particulate matter (SPM), formaldehyde, carbon monoxide (CO), polycyclic fragrant hydrocarbons, nitrogen dioxide (NO_2_), and much more (68). There is growing evidence suggesting that exposure to these pollutants may raise the risk of cardiac events and associated mortality. It is estimated that the use of solid fuels in residences, mostly for cooking, causes more than 3.5 million early deaths per annum and approximately 110 million disability-adjusted life years (DALYs) (69, 70). The effects of indoor air pollution on cardiac health and outcomes have received less attention in emerging Asian nations, although they use equivalent or more biomass fuel than industrialized countries, indicating higher levels of indoor air pollution and relatively higher CVD burdens (61). It is one of the significant worldwide public health challenges and needs increased preventative endeavors *via* studies and legislation. Multiple workarounds are known to lower indoor emissions while cooking. At the community scale, energy technology must be modernized and public knowledge of the severity of cooking-related pollution must increase. Adequate actions to address a wide range of cooking-related difficulties through education, economic growth, and renewable energy resources may be very advantageous in lowering the potential heart health risks caused by biomass fuel emissions.

The data from GBD relied on regularly accessible statistics from several countries and regions, despite being comprehensive and open to almost all nations around the globe. There may be variations in the reliability of data collection and processing and the validity of the cause of mortality. In addition, if the variables (risk factors) are extended, subsequent research may be conducted using more grey relational methodologies to do a comparative analysis with other regions of the world.

## Conclusion

Our insights have considerable implications for health policies and decision-makers for achieving sustainable development goals (SDGs) of wellbeing and good health. To attain a health goal with a perspective of CVD, we need to comprehend that the healthcare industry is merely one of many contributors to healthier longevity. Nutrition, lack of physical activity, environmental sustainability, and tobacco usage will all be impacted by agricultural, climatic, transportation, economic, and trade deals. To endorse and vindicate a meaningful reversion in cardiovascular health, we must cooperate across disciplines and geographic boundaries. Only then could we encourage other organizations and enterprises to devote themselves to our shared objectives, which are essential to global population healthcare and prosperity. Ideally, the SAARC governments should solidly work together, advocate, and emphasize initiatives that might reduce the burden of CVD in the zone *via* evidence-based therapeutics encompassing prompt diagnostics and prevention to optimal care and treatments. We suggest that multi-sectoral cooperation involving all stakeholders is vital to develop an awareness of risk factors, mitigation, treatment, and aftercare of heart disease across the South Asian region.

## Data availability statement

Publicly available datasets were analyzed in this study. This data can be found at: Institute for Health Metrics and Evaluation (IHME) Global Burden of Disease (GBD) 2019, https://www.healthdata.org/gbd/2019.

## Author contributions

Both authors listed have made a substantial, direct, and intellectual contribution to the work and approved it for publication.

## Conflict of interest

The authors declare that the research was conducted in the absence of any commercial or financial relationships that could be construed as a potential conflict of interest.

## Publisher's note

All claims expressed in this article are solely those of the authors and do not necessarily represent those of their affiliated organizations, or those of the publisher, the editors and the reviewers. Any product that may be evaluated in this article, or claim that may be made by its manufacturer, is not guaranteed or endorsed by the publisher.

## References

[1] Lear SA Hu W Rangarajan S Gasevic D Leong D Iqbal R. The effect of physical activity on mortality and cardiovascular disease in 130 000 people from 17 high-income, middle-income, and low-income countries: the PURE study. Lancet. (2017) 390:2643–54. 10.1016/S0140-6736(17)31634-3 28943267

[2] Gheorghe A Griffiths U Murphy A Legido-Quigley H Lamptey P Perel P. The economic burden of cardiovascular disease and hypertension in low-and middle-income countries: a systematic review. BMC Pub Health. (2018) 18:975. 10.1186/s12889-018-5806-x 30081871 PMC6090747

[3] Zhao D Liu J Wang M Zhang X Zhou M. Epidemiology of cardiovascular disease in China: current features and implications. Nat Rev Cardiol. (2019) 16:203–12. 10.1038/s41569-018-0119-4 30467329

[4] W. P. by Country. World population by country. (2020). Available online at: https://worldpopulationreview.com/ (accessed May 16, 2022).

[5] Hussain SM Oldenburg B Wang Y Zoungas S Tonkin AM. (2013). Assessment of cardiovascular disease risk in South Asian populations. Int J Vasc Med. (2013) 2013. 10.1155/2013/786801 24163770 PMC3791806

[6] Rehman S Li X Wang C Ikram M Rehman E Liu M. Quality of care for patients with acute myocardial infarction (AMI) in Pakistan: a Retrospective Study. Int J Environ Res Public Health. (2019) 16:3890. 10.3390/ijerph16203890 31615067 PMC6844119

[7] Cainzos-Achirica M Fedeli U Sattar N Agyemang C Jenum AK McEvoy JW. Epidemiology, risk factors, and opportunities for prevention of cardiovascular disease in individuals of South Asian ethnicity living in Europe. Atherosclerosis. (2019) 286:105–13. 10.1016/j.atherosclerosis.05014 31128454 PMC8299475

[8] Li X Wang C Rehman S Wang X Zhang W Su S. Setting performance benchmarks for stroke care delivery: which quality indicators should be prioritized in quality improvement; an analysis in 500,331 stroke admissions. Int J Stroke. (2020) 16:1747493020958608. 10.1177/1747493020958608 32957865

[9] Rehman S Rehman N Naz M Mumtaz A Jianglin Z. (2021). Application of grey-based SWARA and COPRAS techniques in disease mortality risk assessment. J Healthc Eng. (2021) 2021. 10.1155/2021/7302157 34900200 PMC8654538

[10] Kumar R Pandey AK Baz A Alhakami H Alhakami W Agrawal A. Fuzzy-based symmetrical multi-criteria decision-making procedure for evaluating the impact of harmful factors of healthcare information security. Symmetry. (2020) 12:664. 10.3390/sym12040664

[11] Rehman S Rehman E Hussain I Jianglin Z. (2021). Socioeconomic influence on cardiac mortality in the south asian region: new perspectives from grey modeling and G-TOPSIS. J Healthc Eng. (2021) 2021. 10.1155/2021/6866246 34804456 PMC8598329

[12] Frazão TDC Camilo DGG Cabral ELS Souza RP. Multicriteria decision analysis (MCDA) in health care: a systematic review of the main characteristics and methodological steps. BMC Med Inform Decis Mak. (2018) 18:90. 10.1186/s12911-018-0663-1 30382826 PMC6211490

[13] Sun H Yu F Xu H Xia H. A case-based grey relational analysis model for multiple criteria classification of thyroid nodules. J Grey Syst. (2020) 32:4.

[14] Dehe B Bamford D. Development, test and comparison of two multiple criteria decision analysis (MCDA) models: a case of healthcare infrastructure location. Expert Syst Appl. (2015) 42:6717–27. 10.1016/j.eswa.2015.04.059.

[15] Aung TS Luan S Xu Q. Application of multi-criteria-decision approach for the analysis of medical waste management systems in Myanmar. J Clean Prod. (2019) 222:733–45. 10.1016/j.jclepro.2019.03.049.

[16] Etim MA Academe S Emenike P Omole D. Application of multi-criteria decision approach in the assessment of medical waste management systems in Nigeria. Sustainability. (2021) 13:10914. 10.3390/su131910914

[17] Agrawal A Pandey AK Baz A Alhakami H Alhakami W Kumar R. Evaluating the security impact of healthcare Web applications through fuzzy based hybrid approach of multi-criteria decision-making analysis. IEEE Access. (2020) 8:135770–83. 10.1109/ACCESS.2020.3010729

[18] Biswas S. Measuring performance of healthcare supply chains in India: a comparative analysis of multi-criteria decision making methods. Decis Mak Appl Manag Eng. (2020) 3:162–89. 10.31181/dmame2003162b

[19] Ozsahin I Abebe ST Mok GS. A multi-criteria decision-making approach for schizophrenia treatment techniques. Arch Psychiatry Psychother. (2020) 22:52–61. 10.12740/APP/111624

[20] Sayan M Sarigul Yildirim F Sanlidag T Uzun B Uzun Ozsahin D Ozsahin I. (2020). Capacity evaluation of diagnostic tests for COVID-19 using multicriteria decision-making techniques. Comput Math Methods Med. (2020) 2020. 10.1155/2020/1560250 32802146 PMC7411452

[21] Javed SA Liu S Mahmoudi A Nawaz M. Patients' satisfaction and public and private sectors' health care service quality in Pakistan: Application of grey decision analysis approaches. Int J Health Plann Manage. (2019) 34:e168–82. 10.1002/hpm.2629 30160783

[22] Hancerliogullari G Hancerliogullari KO Koksalmis E. The use of multi-criteria decision making models in evaluating anesthesia method options in circumcision surgery. BMC Med Inform Decis Mak. (2017) 17:1–13. 10.1186/s12911-017-0409-5 28114944 PMC5260115

[23] Bharsakade RS Acharya P Ganapathy L Tiwari MK. A lean approach to healthcare management using multi criteria decision making. Opsearch. (2021) 58:1–26. 10.1007/s12597-020-00490-5

[24] Ozsahin I. Identifying a personalized anesthetic with fuzzy PROMETHEE. Healthc Inform Res. (2020) 26:201–11. 10.4258/hir.2020.26.3.201 32819038 PMC7438688

[25] Yildirim FS Sayan M Sanlidag T Uzun B Ozsahin DU Ozsahin I. (2021). Comparative evaluation of the treatment of COVID-19 with multicriteria decision-making techniques. J Healthc Eng. (2021) 2021. 10.1155/2021/8864522 33552457 PMC7831275

[26] Fei L Lu J Feng Y. An extended best-worst multi-criteria decision-making method by belief functions and its applications in hospital service evaluation. Comput Ind Eng. (2020) 142:106355. 10.1016/j.cie.2020.106355

[27] Ozsahin I Ozsahin DU Nyakuwanikwa K Simbanegavi TW. “Fuzzy PROMETHEE for ranking pancreatic cancer treatment techniques,” in *2019 Advances in Science and Engineering Technology International Conferences (ASET)*. Dubai (2019), p. 1–5.

[28] Sharma M Sehrawat R. A hybrid multi-criteria decision-making method for cloud adoption: evidence from the healthcare sector. Technol Soc. (2020) 61:101258. 10.1016/j.techsoc.2020.101258

[29] Ozsahin DU Uzun B Musa MS Sentürk N Nurçin FV Ozsahin I. Evaluating nuclear medicine imaging devices using fuzzy PROMETHEE method. Procedia Comput Sci. (2017) 120:699–705. 10.1016/j.procs.11.298.

[30] La Fata CM Lupo T Piazza T. Service quality benchmarking via a novel approach based on fuzzy ELECTRE III and IPA: an empirical case involving the Italian public healthcare context. Health Care Manag Sci. (2019) 22:106–20. 10.1007/s10729-017-9424-4 29164424

[31] Vimal KEK Kandasamy J Nadeem SP Kumar A Šaparauskas J Garza-Reyes JA. Developing a strategic sustainable facility plan for a hospital layout using ELECTRE and Apples procedure. Int J Strateg Prop Manag. (2021) 25:17–33. 10.3846/ijspm.2020.13733

[32] Quartey-Papafio TK Islam S Dehaghani AR. Evaluating suppliers for healthcare centre using ordinal priority approach. Manag Sci Bus Decis. (2021) 1:5–11. 10.52812/msbd.12

[33] Ng DKW Deng J. Contrasting grey system theory to probability and fuzzy. ACM Sigice Bull. (1995) 20:3–9. 10.1145/202081.202082

[34] Liu S Xie N Forrest J. Novel models of grey relational analysis based on visual angle of similarity and nearness. Grey Syst Theory Appl. (2011) 1:8–18. 10.1108/20439371111106696

[35] Rehman E Rehman S. Modeling the nexus between carbon emissions, urbanization, population growth, energy consumption, and economic development in Asia: Evidence from grey relational analysis. Energ Rep. (2022) 8:5430–42. 10.1016/j.egyr.03.179.

[36] Javed SA. A Novel Research on Grey Incidence Analysis Models and Its Application in Project Management. Nanjing University of Aeronautics and Astronautics: China (2019).

[37] Feng SJ Ma YD Song ZL Ying J. Forecasting the energy consumption of China by the grey prediction model. Energ Sour Part B Econ Plann Policy. (2012) 7:376–89. 10.1080/15567240903330426

[38] Rehman S Rehman E Mumtaz A Jianglin Z. A Multicriteria decision-making approach in exploring the nexus between wind and solar energy generation, economic development, fossil fuel consumption, and CO_2_ emissions. Front Environ Sci. (2022) 659: 819384. 10.3389/fenvs.2021.819384

[39] Mumtaz A Rehman E Rehman S Hussain I. Impact of environmental degradation on human health: an assessment using multicriteria decision making. Front. Public Heal. (2021) 9: 812743. 10.3389/fpubh.2021.812743 35127627 PMC8810485

[40] Kalyon A Günay M Özyürek D. Application of grey relational analysis based on Taguchi method for optimizing machining parameters in hard turning of high chrome cast iron. Adv Manuf. (2018) 6:419–29. 10.1007/s40436-018-0231-z

[41] Dhungana RR Pandey AR Shrestha N. Trends in the prevalence, awareness, treatment, and control of hypertension in Nepal between 2000 and 2025: a systematic review and meta-analysis. Int J Hypertens. (2021) 2021. 10.1155/2021/6610649 33747559 PMC7952181

[42] Gupta R Xavier D. (2018). Hypertension: the most important non communicable disease risk factor in India. Indian Heart J. (2018) 70:565–72. 10.1016/j.ihj.2018.02.003. 30170654 PMC6116711

[43] Walsh EI Jacka FN Butterworth P Anstey KJ Cherbuin N. The association between Western and Prudent dietary patterns and fasting blood glucose levels in type 2 diabetes and normal glucose metabolism in older Australian adults. Heliyon. (2017) 3:e00315. 10.1016/j.heliyon.2017.e00315 28626807 PMC5466591

[44] Fatema K Zwar NA Milton AH Ali L Rahman B. Prevalence of risk factors for cardiovascular diseases in Bangladesh: a systematic review and meta-analysis. PLoS One. (2016) 11:e0160180. 10.1371/journal.pone.0160180 27494706 PMC4975457

[45] Raghavan S Vassy JL Ho YL Song RJ Gagnon DR Cho K. Diabetes mellitus–related all-cause and cardiovascular mortality in a national cohort of adults. J Am Heart Assoc. (2019) 8:e011295. 10.1161/JAHA.118.011295 30776949 PMC6405678

[46] Ortega FB Lavie CJ Blair SN. Obesity and cardiovascular disease. Circ Res. (2016) 118:1752–70. 10.1161/CIRCRESAHA.115.306883 27230640

[47] Han TS Lean MEJ. A clinical perspective of obesity, metabolic syndrome and cardiovascular disease. JRSM Cardiovasc Dis. (2016) 5:2048004016633371. 10.1177/2048004016633371 26998259 PMC4780070

[48] Mandviwala T Khalid U Deswal A. Obesity and cardiovascular disease: a risk factor or a risk marker? Curr Atheroscler Rep. (2016) 18:21. 10.1007/s11883-016-0575-4 26973130

[49] Ghoorah K Campbell P Kent A Maznyczka A Kunadian V. Obesity and cardiovascular outcomes: a review. Eur Hear J Acute Cardiovasc Care. (2016) 5:77–85. 10.1177/2048872614523349 24526749

[50] Nepal G Tuladhar ET Dahal S Ahamad ST Adhikari S Kandel A. Lifestyle practices and obesity in Nepalese youth: a cross-sectional study. Cureus. (2018) 10:2. 10.7759/cureus.2209 29686951 PMC5910009

[51] Vaidya A Krettek A. Physical activity level and its sociodemographic correlates in a peri-urban Nepalese population: a cross-sectional study from the Jhaukhel-Duwakot health demographic surveillance site. Int J Behav Nutr Phys Act. (2014) 11:1–12. 10.1186/1479-5868-11-39 24628997 PMC3984675

[52] Dhungana RR Devkota S Khanal MK Gurung Y Giri RK Parajuli RK. Prevalence of cardiovascular health risk behaviors in a remote rural community of Sindhuli district, Nepal. BMC Cardiovasc Disord. (2014) 14:1–8. 10.1186/1471-2261-14-92 25066117 PMC4115072

[53] Banks E. Tobacco smoking and risk of 36 cardiovascular disease subtypes: fatal and non-fatal outcomes in a large prospective Australian study. BMC Med. (2019) 17:1–18. 10.1186/s12916-019-1351-4 31266500 PMC6607519

[54] Kondo T Nakano Y Adachi S Murohara T. Effects of tobacco smoking on cardiovascular disease. Circ J. (2019) 83:1980–5. 10.1253/circj.CJ-19-0323 31462607

[55] Mishra S Joseph RA Gupta PC Pezzack B Ram F Sinha DN. Trends in bidi and cigarette smoking in India from 1998 to 2015, by age, gender and education. BMJ Glob Heal. (2016) 11:e000005. 10.1136/bmjgh-2015-000005 28588906 PMC5321300

[56] Wang Y Lu Z Zhang J Yang Y Shen J Zhang X. The APOA5 rs662799 polymorphism is associated with dyslipidemia and the severity of coronary heart disease in Chinese women. Lipids Health Dis. (2016) 15:1–9. 10.1186/s12944-016-0343-z 27716220 PMC5054624

[57] Zhang FL Xing YQ Wu YH Liu HY Luo Y Sun MS. The prevalence, awareness, treatment, and control of dyslipidemia in northeast China: a population-based cross-sectional survey. Lipids Health Dis. (2017) 16:1–13. 10.1186/s12944-017-0453-2 28330492 PMC5363017

[58] Basit A Sabir S Riaz M Fawwad A. NDSP 05: Prevalence and pattern of dyslipidemia in urban and rural areas of Pakistan; a sub analysis from second National Diabetes Survey of Pakistan (NDSP) 2016–2017. J Diabetes Metab Disord. (2020) 19:1–11. 10.1007/s40200-020-00631-z 33520835 PMC7843689

[59] Naeem N Butt A Bin Zafar A Fawwad A Tahir B Basit A. Dyslipidemia pattern among newly diagnosed and known type 2 diabetics: a comparative analysis from a tertiary care hospital of Karachi, Pakistan. Pak J Med Res. (2020) 59:45–50.

[60] Rehman S Rehman E Mumtaz A Jianglin Z. Cardiovascular disease mortality and potential risk factor in china: a multi-dimensional assessment by a grey relational approach. Int J Public Health. (2022) 67:1604599. 10.3389/ijph.2022.1604599 35574277 PMC9101313

[61] Yamamoto SS Phalkey R Malik AA. (2013). A systematic review of air pollution as a risk factor for cardiovascular disease in South Asia: limited evidence from India and Pakistan. Int J Hyg Environ Health. (2014) 217:133–44. 10.1016/j.ijheh.2013.08.003. 24064368

[62] Prasad D. Operation Research. Oxford: Alpha Science International Limited (2015).

[63] Wen M Iwamura K. Fuzzy facility location-allocation problem under the Hurwicz criterion. Eur J Oper Res. (2006) 184:627–35. 10.1016/j.ejor.2006.11.029.

[64] Abdul-Aziz AA Desikan P Prabhakaran D Schroeder LF. Tackling the burden of cardiovascular diseases in India: the essential diagnostics list. Circ Cardiovasc Qual Outcomes. (2019) 12:e005195. 10.1161/CIRCOUTCOMES.118.005195 30917685

[65] Prabhakaran D Jeemon P Roy A. Cardiovascular diseases in India: current epidemiology and future directions. Circulation. (2016) 133:1605–20. 10.1161/CIRCULATIONAHA.114.008729 27142605

[66] Rehman S Rehman E Ikram M Jianglin Z. Cardiovascular disease (CVD): assessment, prediction and policy implications. BMC Public Health. (2021) 21:1–14. 10.1186/s12889-021-11334-2 34215234 PMC8253470

[67] Meo SA Suraya F. Effect of environmental air pollution on cardiovascular diseases. Eur Rev Med Pharmacol Sci. (2015) 19:4890–7. 26744881

[68] Clark ML Peel JL Balakrishnan K Breysse PN Chillrud SN Naeher LP. Health and household air pollution from solid fuel use: the need for improved exposure assessment. Environ Health Perspect. (2013) 121:1120–8. 10.1289/ehp.1206429 23872398 PMC3801460

[69] Vidale S Campana C. Ambient air pollution and cardiovascular diseases: from bench to bedside. Eur J Prev Cardiol. (2018) 25:818–25. 10.1177/2047487318766638 29580063

[70] Mumtaz A Rehman N Haider A Rehman S. Long-term air pollution exposure and ischemic heart disease mortality among elderly in highly aged Asian economies. Front Public Heal. (2022) 2337:819123. 10.3389/fpubh.2021.819123 35198535 PMC8860192

